# Which is the best in carbamazepine overdose?

**DOI:** 10.1002/ccr3.1118

**Published:** 2017-08-22

**Authors:** Kıvanç Karaman, Kenan Ahmet Türkdoğan, Ahmet Tunç Deniz, Selçuk Eren Çanakçı

**Affiliations:** ^1^ Emergency Service Sinop Ataturk State Hospital Sinop Turkey; ^2^ Department of Emergency Medicine Adnan Menderes University Aydin Turkey

**Keywords:** Carbamazepine, extracorporeal treatments, intoxication, intravenous lipid emulsion

## Abstract

Intravenous lipid emulsion treatment is safer, faster, and easier to apply and could be a powerful alternative to extracorporeal treatment methods in carbamazepine intoxication.

## Introduction

Carbamazepine has a structure similar to that of tricyclic antidepressants and is used for the treatment of bipolar disorder, neuropathic pain, hyperactivity, and seizure disorder. It is highly lipophilic, highly bound to plasma proteins and distributes rapidly and extensively (volume of distribution ranges from 0.8 to 1.4 L/kg). Toxicity from Carbamazepine overdose was first described in 1967 and continues to be responsible for a large proportion of life‐threatening cases among anticonvulsant poisonings 1. Neurologic, respiratory, and cardiac findings such as altered mental status, seizures, respiratory depression, concomitant aspiration, tachycardias, hypotension, and even death could be seen in Carbamazepine intoxication 2, 3. The therapeutic concentration range is 4–12 mg/L (17–51 *μ*mol/L); significant toxicity usually occurs over 40 mg/L (169 *μ*mol/L), but also potentially at lower concentrations 3, 4. According to blood Carbamazepine levels, poisoning can be classified into four stages: potentially catastrophic relapse with levels <11 mg/L, disorientation and ataxia at levels of 11–15 mg/L, combativeness and hallucinations at levels of 15–25 mg/L, and convulsions and coma at levels >25 mg/L 5. Multiple‐dose activated charcoal (MDAC) increases elimination and improves clinical outcome in patients with Carbamazepine overdose, and is recommended for patients with life‐threatening ingestions 6, 7. Extracorporeal treatments (ECTRs) are suggested if prolonged coma or respiratory depression requiring mechanical ventilation is present or expected 3. Additionally some case reports have described successful treatment of Carbamazepine toxicity with intravenous lipid emulsion (ILE) therapy in recent years 8, 9.

## Case Report

A 35‐year‐old man was brought to our service 5 h after ingestion of 100 tablets Carbamazepine (each tablet contains 400 mg controlled‐release Carbamazepine). On admission, his blood pressure was 90/60 mmHg, heart rate was 96 bpm, respiratory rate was 19 bpm, and Glasgow Coma Scale (GKS) was 7. Because he was unconscious, he was entubated and transferres to intensive care unit (ICU) immediately. Gastric lavage was performed, and activated charcoal was given. Simultaneous blood carbamazepine level was studied in patients with normal laboratory values. Carbamazepine level on admission was 70.8 mg/L (Table 1). Hemoperfusion (HP), hemodialysis (HD), ILE, and MDAC treatments were planned. A 135 mL bolus and 375 mL for the first hour followed by 25 mL/h infusion of ILE was administered to the patient until the carbon filter (Adsorba 300C) was provided to start HP therapy. At the 3rd hour of admission, ILE treatment was interrupted and HP and HD protocol started. However, due to obstruction of the ultrafiltrate, 2‐h HP (1 m^2^ dialyzer “Adsorba 300C” at a flow rate of 250 mL/min) and 1‐h HD (with FX50 filter, 250 mL/min flow rate, 500 cc dialysate and 1000 units heparin infused HD) could be performed to the patient. After HP and HD treatments, blood carbamazepine level decreased to 58.7 mg/L, and 25 mL/h dose of ILE infusion was started again. Patient's platelet count decreased to 98,000 after first HP and HD treatments (Table 1). Thrombocytopenia was thought to be attributed to HP and no medication applied. Between 18th and 25th hours of patient's follow‐up, 3 h of HP and 3 h of HD were performed; 2000 cc Ultrafiltration was made during the HD. ILE treatment was interrupted during HP, HD protocols. At the 2nd hour of the HP session, the patient opened his eyes spontaneously (GCS: 11). After HP and HD treatments 25 mL/h dose of ILE infusion was started again. Patient's platelet count decreased by 116,000–59,000 and blood carbamazepine level decreased to 39.6 mg/L after second HP and HD treatments. Between 24th and 48th hours of patient's follow‐up, ILE treatment continued at 25 mL/h dose and no further HP or HD administration was applied. At the 48th hour of patient's follow‐up, ILE and MDAC treatments were also stopped, and patient was extubated due to patient's GCS was 15, serum carbamazepine level was 17.7 mg/L and had spontaneous respiration. Patient discharged from ICU to emergency observation unit at the 72nd hour of follow‐up with 10.6 mg/L blood carbamazepine level and 95,000 platelet count due to his general condition and vital signs were stable. His last blood carbamazepine level was 4.3 mg/L, and platelet count was 220,000 at the 96th hour of follow‐up so that patient transferred to Psychiatric Unit.

**Table 1:** Changes in the patient's GKS, laboratory tests, and treatment

Time	Carbamazepine level (mg/L)	GKS	Trombosit (/mcrL)	Treatments
Admission	70.8	7	186,000	Bolus ILE, AC, Entubation, Transferred to ICU
0–6 h	58.7	7	98,000	2 h HP, 1 h HD, 3 h ILE infusion, second dose of AC
6–18 h	58.7	7	116,000	12 h ILE infusion, 2 dose AC
18–24 h	39.6	11	59,000	3 h HP, 3 h HD, 5th dose of AC
24–48 h	17.7	15	61,000	24 h ILE, 4 dose AC, Extubation
48–72 h	10.6	15	95,000	Transferred to Observation Unit, Conservative treatments
72–96 h	4.3	15	220,000	Transferred to Psychiatric Unit

## Discussion

Carbamazepine is mainly metabolized in the liver. It is highly lipophilic, highly bound to plasma proteins (80–85%) and distributes rapidly and extensively. Clinical findings in overdose are related to amount of ingestion and blood Carbamazepine level. Coma, hypoventilation, arrhythmias, hemodynamic instability, and even death can occur in Carbamazepine intoxication. Although death is unusual, it has been reported in a cohort study that overall mortality was 13%; mean Carbamazepine ingestion in lethal cases was 23.6 g 2, 10. In our case, ingestion is 40 g, and initial blood Carbamazepine level is 70.8 mg/L. The patient entubated in the early period and aggressive treatments planned because this amount was above the level which serious toxic findings are seen.

The effects of carbamazepine overdose are often substantially prolonged, compared to the effects of therapeutic doses, and patients may have reduced conscious level for up to several days. In overdose situations, carbamazepine can delay its own absorption by reducing gastrointestinal system motility with anticholinergic effect, which is more pronounced in slow release tablet forms. MDAC increases elimination and improves clinical outcome in patients with Carbamazepine overdose and is recommended for patients with life‐threatening ingestions 6, 7. Therefore, orogastric lavage performed, MDAC started and continued for 48 h, although 5 h past after ingestion.

Extracorporeal treatment is suggested if prolonged coma or respiratory depression requiring mechanical ventilation is present or expected 3. Although their effect on mortality is not fully disclosed, it is stated that ECTR methods reduce the need for mechanical ventilation, intensive care stay, and associated costs in Carbamazepine overdose 11. Although many ECTR methods have been tried in the treatment of carbamazepine intoxication, the suggested methods are HP and HD 3. It is widely believed that HP is more effective in drug elimination because of the high protein‐binding ratio but in the recent years with using high‐efficiency high‐flux membranes, HD efficity has been reported to be as high as HP 12, 13, 14. It is also reported in recent years that ILE treatment is effective for carbamazepine intoxication 8, 9, 15.

Considering this information, we combined HP, HD, and ILE treatments in the first 24 h to have a rapid effect in our patient with high serum carbamazepine level and severe toxicity findings. In the first 6 h of admission, in addition to ILE treatment, 2 h of HP and 1 h of HD were applied to patient and serum carbamazepine level decreased by 12% to 58.7 mg/L at 6th hour. Then 12 h later, 3‐h HP and 3‐h HD were applied to patient again and serum carbamazepine level decreased by 33% to 39.6 mg/L. So that at the end of the first 24 h the reduction of serum carbamazepine level is 55% with combined therapy. On the other hand, patient got only ILE therapy on second day and Carbamazepine level decrease by 44%.

In a case report presented by Kahveci et al., in which HP and HD treatments were applied together in a similar way to our case, after administration 3‐h HP and 4‐h HD blood carbamazepine level decreased from 46.5 to 4.9 mg/L. In this case, blood carbamazepine level decreased by 57% with HP, 75% with HD 12. Combined HD and HP resulted in a 50% reduction in carbamazepine levels in a study by Bock et al. 16.Efficacy of HP in reducing plasma levels of carbamazepine range from 20% to 50%^14^. High‐efficiency HD when used alone is also effective in decreasing drug levels based on several case reports 13, 14, 17. Compared with the literature, the decrease in serum carbamazepine level in our case is expected with the combined treatment on the first day. The reason of that might be short duration of the first HP and HD session due to obstruction of ultrafiltration. It is also conceivable that the initial bolus ILE treatment may have caused clogging of the ultrafiltrate. As a matter of fact, there was a higher decrease in serum carbamazepine level after the second HP and HD session. Nevertheless, the effect of combined HP, HD, and ILE treatments is under the expectation.

In 1998, Weinberg defined the widely accepted “lipid sink” theory to explain the mechanism of elimination of lipid soluble drugs. After reports in local anesthetic toxicity by Rosenblatt et al., ILE was began to use in other lipophilic drug intoxications such as beta blockers, calcium channel blockers, and several psychotropic agents 18, 19. ILE is most effective in treating cardiotoxicity related to anesthetic agents, whereas its role for nonanesthetic agents has given very mixed outcomes in published cases 20. Carbamazepine is a molecule with a high lipid solubility, but there are very little data in the literature to show the benefits of ILE treatment in carbamazepine intoxication, and these are limited to case presentations. In two case reports, it was stated that ILE treatment may be useful for carbamazepine‐induced cardiotoxicity 8, 15. In a case with severe carbamazepine toxication published by Avcil et al. also showed a 42% reduction in serum carbamazepine level by 24 h with ILE administration without ECTR methods 9. This result coincides with the outcome of our case. But compared to our patient that patient has a lower serum carbamazepine level and a higher GKS score. There was also an increase in the QT distance due to carbamazepine intake in that patient, and ILE treatment was chosen because of cardiotoxicity. In our case, no carbamazepine‐related cardiac findings were detected.

Despite the fact that the level of carbamazepine was within the normal range and the clinical situation was appropriate for discharge at the 3rd day of follow‐up, the patient continued to follow for thrombocytopenia and could be discharged on the 4th day. The only complication, seen in follow‐up, was thrombocytopenia which is attributed to HP treatment. Complications due to ILE, such as fat overload syndrome, hypervolemia, hyperamylasemia, hemolysis, icterus, seizures, and increased clotting time, were not seen in our patient 21, 22. In addition, although ILE therapy was started immediately in the patient's admission, we had to wait for the filter to begin HP treatment. This suggests that ILE treatment is safer, faster, and easier to apply than ECTR methods.

We could not find any report have been found on the concurrent application of ECTR methods and ILE treatment in carbamazepine intoxication in the literature. Our report is the first to compare the effects of using ECTR methods and ILE treatment together. In conclusion, we think that ILE treatment is as effective as ECTR methods like HP, HD even at high serum concentrations in carbamazepine intoxication, besides ILE treatment is safer, easier, and quicker to apply. Nevertheless, we conclude that the combined use of ILE therapy and ECTR methods does not provide additional benefit in clinical practice. While acknowledging that this outcome we obtained with a single‐case presentation is not sufficient, and further research is necessary, we believe that ILE treatment is a powerful alternative to ECTR methods in carbamazepine intoxication.

## Authorship

KK: was responsible for the hospitalization and outpatient follow‐up of the case and also had the major role in writing the manuscript. KAT: Director of the Emergency Medicine department, is responsible for the hospitalization and outpatient follow‐up of the case. ATD: resident doctor, is responsible for patient's hospitalization. SEÇ: resident doctor, is responsible for patient's hospitalization. All authors had access to the data and a role in writing the manuscript.

## Conflict of Interest

None declared.

## References

[1] Guntelberg, E. 1967 Carbamazepine tegretol poisoning. A review and case report. Ugeskr. Laeger 129:161–163. 5602757

[2] Schmidt, S. , and M. Schmitz‐Buhl . 1995 Signs and symptoms of carbamazepine overdose. J. Neurol. 242:169–173. 7751861 10.1007/BF00936891

[3] Ghannoum, M. , C. Yates , T. F. Galvao , K. M. Sowinski , T. H. Vo , A. Coogan , et al. 2014 Extracorporeal treatment for carbamazepine poisoning: systematic review and recommendations from the EXTRIP workgroup. Clin. Toxicol. 52:993–1004. 10.3109/15563650.2014.973572 PMC4782683 25355482

[4] Hojer, J. , H. O. Malmlund , and A. Berg . 1993 Clinical features in 28 consecutive cases of laboratory confirmed massive poisoning with carbamazepine alone. J. Toxicol. Clin. Toxicol. 31:449–458. 8355320 10.3109/15563659309000412

[5] Unei, H. , H. Ikeda , T. Murakami , K. Tanigawa , and K. Kihira . 2008 Detoxication treatment for carbamazepine and lithium overdose. Yakugaku Zasshi 128:165–170. 18176069 10.1248/yakushi.128.165

[6] Brahmi, N. , N. Kouraichi , H. Thabet , and M. Amamou . 2006 Influence of activated charcoal on the pharmacokinetics and the clinical features of carbamazepine poisoning. Am. J. Emerg. Med. 24:440–443. 16787802 10.1016/j.ajem.2005.12.025

[7] Vale, J. , E. P. Krenzelok , and V. D. Barceloux . 1999 Position statement and practice guidelines on the use of multi‐dose activated charcoal in the treatment of acute poisoning. American Academy of Clinical Toxicology; European Association of Poisons Centres and Clinical Toxicologists. J. Toxicol. Clin. Toxicol. 37:731–751. 10584586 10.1081/clt-100102451

[8] Sud, A. , and K. Chidley . 2013 A role for intravenous lipid emulsion in cardiac arrest secondary to life‐threatening carbamazepine overdose. Anaesthesia 68:989.

[9] Avcil, M. , Y. E. Ozluer , K. Karaman , M. Kapcı , and B. Kantekın . 2015 Treatment of Severe Carbamazepine Intoxication with Intravenous Lipid Emulsion Therapy. J. Pharmacol. Clin. Toxicol. 3:1052.

[10] Spiller, H. A. , E. P. Krenzelok , and E. Cookson . 1990 Carbamazepine overdose: a prospective study of serum levels and toxicity. J. Toxicol. Clin. Toxicol. 28:445–458. 2269999 10.3109/15563659009038587

[11] Askenazi, D. J. , S. L. Goldstein , I. F. Chang , E. Elenberg , and D. I. Feig . 2004 Management of a severe carbamazepine overdose using albumin enhanced continuous venovenous hemodialysis. Pediatrics 113:406–409. 14754959 10.1542/peds.113.2.406

[12] Kahveci, A. , Z. Kubilay , E. Aşıcıoğlu , H. Arıkan , M. Koç , S. Tuğlular ve Ark , et al. 2011 Karbamazepin İntoksikasyonunda Karbon Hemoperfüzyon mu Yüksek Etkinlikli Hemodiyaliz Uygulaması mı?: Bir Olgu Sunumu. Turk. Neph. Dial. Transpl. 20:192–194.

[13] Tapolyai, M. , M. Campbell , K. Dailey , and S. Udvari‐Nagy . 2002 Hemodialysis is as effective as hemoperfusion for drug removal in carbamazepine poisoning. Nephron 90:213–215. 11818708 10.1159/000049045

[14] Kielstein, J. T. , A. Schwarz , A. Arnavaz , O. Sehlberg , H. M. Emrich , and D. Fliser . 2002 High‐flux hemodialysis–an effective alternative to hemoperfusion in the treatment of carbamazepine intoxication. Clin. Nephrol. 57:484–486. 12078955 10.5414/cnp57484

[15] Hurley, W. T. , and P. Hanlon . 2009 Lipid emulsion as an antidote at the Washington poison center; use in carbamazepine, flecanide, hydroxychloroquine, Bupivacaine and Bupropion. Clin. Toxicol. 47:729.

[16] Bock, E. , F. Keller , J. Heitz , and G. Heinmeyer . 1989 Treatment of carbamazepine poisoning by combined hemodialysis/hemoperfusion. Int. J. Clin. Pharm. Ther. Toxicol. 27:490–492. 2684869

[17] Koh, K. H. , and H. H. Tan . 2006 High‐flux haemodialysis treatment as treatment for carbamazepine intoxication. Med. J. Malaysia 61:109–111. 16708747

[18] Rosenblatt, M. A. , M. Abel , G. W. Fischer , C. J. Itzkovich , and J. B. Eisenkraft . 2006 Successful use of a 20% lipid emulsion to resuscitate a patient after a presumed bupivacaine‐related cardiac arrest. Anesthesiology 105:217–218. 16810015 10.1097/00000542-200607000-00033

[19] Weinberg, G. L. 2012 Lipid emulsion infusion: resuscitation for local anesthetic and other drug overdose. Anesthesiology 117:180–187. 22627464 10.1097/ALN.0b013e31825ad8de PMC4609208

[20] Waring, W. S. , et al. 2012 Intravenous lipid administration for drug‐induced toxicity: a critical review of the existing data. Expert Rev. Clin. Pharmacol. 5:437–444. 22943123 10.1586/ecp.12.27

[21] Hojsak, I. , and S. Kolaček . 2014 Fat overload syndrome after the rapid infusion of SMOFlipid emulsion. JPEN J. Parenter. Enteral Nutr. 38:119–121. 23520135 10.1177/0148607113482001

[22] Goulet, O. , R. Girot , M. Maier‐Redelsperger , D. Bougle , J. L. Virelizier , and C. Ricour . 1986 Hematologic disorders following prolonged use of intravenous fat emulsions in children. JPEN J. Parenter. Enteral Nutr. 10:284–288. 3086587 10.1177/0148607186010003284

